# Genome analysis and heterologous expression of acetate-activating enzymes in the anammox bacterium *Kuenenia stuttgartiensis*

**DOI:** 10.1007/s00203-012-0829-7

**Published:** 2012-07-01

**Authors:** Lina Russ, Harry R. Harhangi, Jeroen Schellekens, Bram Verdellen, Boran Kartal, Huub J. M. Op den Camp, Mike S. M. Jetten

**Affiliations:** Department of Microbiology, IWWR, Radboud University Nijmegen, Heyendaalseweg 135, 6525 AJ Nijmegen, The Netherlands

**Keywords:** Acetate, Propionate, Acetyl-coenzyme A, Anammox, AMP

## Abstract

Anaerobic ammonium-oxidizing bacteria were recently shown to use short-chain organic acids as additional energy source. The AMP-forming acetyl-CoA synthetase gene (*acs*) of *Kuenenia stuttgartiensis*, encoding an important enzyme involved in the conversion of these organic acids, was identified and heterologously expressed in *Escherichia coli* to investigate the activation of several substrates, that is, acetate, propionate and butyrate. The heterologously expressed ACS enzyme could complement an *E. coli* triple mutant deficient in all pathways of acetate activation. Activity was observed toward several short-chain organic acids, but was highest with acetate. These properties are in line with a mixotrophic growth of anammox bacteria. In addition to *acs*, the genome of *K. stuttgartiensis* contained the essential genes of an acetyl-CoA synthase/CO dehydrogenase complex and genes putatively encoding two isoenzymes of archaeal-like ADP-forming acetyl-CoA synthetase underlining the importance of acetyl-CoA as intermediate in the carbon assimilation metabolism of anammox bacteria.

**Electronic supplementary material:**

The online version of this article (doi:10.1007/s00203-012-0829-7) contains supplementary material, which is available to authorized users.

## Introduction

Bacteria capable of anaerobic ammonium oxidation (anammox) derive their energy for growth from the conversion of ammonium and nitrite into dinitrogen gas, thereby constituting a significant sink for fixed nitrogen under anoxic conditions (Arrigo 2005; Lam and Kuypers 2011). Cellular carbon is hypothesized to be fixed via the acetyl-coenzyme A (CoA) pathway, suggesting a chemolithoautotrophic lifestyle (Schouten et al. 2004; Strous et al. 2006). Throughout this pathway, organic carbon is formed by reducing CO_2_ to CO and subsequently to cellular components via acetyl-CoA. Interestingly, it was shown recently that anammox bacteria have a more versatile metabolism than previously assumed: several genera were able to oxidize organic compounds to CO_2_ with nitrate and/or nitrite as electron acceptor, possibly refixing the CO_2_ via the acetyl-CoA pathway and fueling the catabolic reaction with nitrite (Güven et al. 2005; Kartal et al. 2007b, 2008). Although the nitrate reduction pathway has been elucidated (Kartal et al. 2007a), the underlying biochemical pathway for organic acid oxidation is still unknown. The abundance of genes potentially involved in organic acid conversion points to its importance in anammox metabolism.

The metabolism of acetate is commonly initiated by its activation to acetyl-CoA that is an essential intermediate of various anabolic and catabolic pathways and has a central role in the carbon metabolism in all three domains of life (Wolfe 2005; Ingram-Smith et al. 2006a). At least five different ways to synthesize acetyl-CoA are known at present (AMP-forming acetyl-CoA synthetase (ACS), ADP-forming acetyl-CoA synthetase (ACD), acetate kinase/phosphotransacetylase (ACKA and PTA), CO dehydrogenase/acetyl-CoA synthase (CODH/ACS) and acetate-CoA transferase). One of these enzyme complexes, an AMP-forming acetyl-CoA synthetase, is essential for the synthesis and conversion of acetate to acetyl-CoA and was experimentally investigated in this study.

ACS catalyzes the direct formation of acetyl-CoA from acetate, ATP and CoA and is present in nearly all organisms. In prokaryotes, it is known to operate often in an assimilatory route during growth on low acetate concentrations (≤10 mM) (Wolfe 2005). It is a member of a family of AMP-forming enzymes that catalyze two-step reactions in which an acyl-adenylate intermediate is formed and pyrophosphate is released (Starai and Escalante-Semerena 2004).

The analysis of the genome sequence of the anammox bacterium *Kuenenia stuttgartiensis* revealed several open reading frames (ORFs) with similarity ≥30 % to known acetate-activating enzymes. Their presence gave a first indication about the route of acetate utilization in anammox bacteria, although the incorporation of acetate-derived carbon into cellular biomass could not be detected so far (Kartal et al. 2007b, 2008).

The present study focused on the functional expression of a putative AMP-forming ACS, the most abundant acetate-activating enzyme in the proteome of *K. stuttgartiensis* encoded in ORF kustc1128 (Kartal et al. 2011). An *ackA*-*pta*-*acs* triple mutant of *E. coli* was complemented with the *K. stuttgartiensis* *acs* gene resulting in recovery of growth on acetate. To investigate the substrate specificity and kinetic properties of the putative ACS, the *acs* gene was overexpressed in the host *E*. *coli* Rosetta™ 2. The potential physiological role of acetate conversion in vivo was determined by colorimetric determination of acetyl-CoA formed from acetate by *K. stuttgartiensis*. This is the first time that an anammox enzyme could be functionally expressed in a heterologous host and that its properties could support an important role in the carbon assimilation metabolism of *K. stuttgartiensis*.

## Materials and methods

### Identification of putative acetate-activating enzymes

To identify gene orthologs of acetate-activating enzymes in the genome of *Kuenenia stuttgartiensis* (Strous et al. 2006), a local BlastX search (National Center for Biotechnology Information, Bethesda) was performed using the *acs* sequence of *Escherichia coli*, *Methanosaeta concilii* and *Saccharomyces cerevisiae*, the *acdA* and *acdB* sequences of *Archaeoglobus fulgidus*, and *ack*-*pta* genes of *E. coli* and *Methanosarcina barkeri* as queries.

### Preparation of DNA and construction of complementation vectors

*Kuenenia stuttgartiensis* biomass was enriched in a sequencing batch reactor (SBR) under conditions commonly used for anammox bacteria (Strous et al. 1998). Cells were harvested by centrifugation at 6,000 rpm for 10 min, resuspended in 20 mM HEPES buffer (pH 7) and disrupted in a French pressure cell. DNA was extracted as described before (Juretschko et al. 1998) and subsequently served as template for a Phusion-based high fidelity PCR (Finnzymes, Finland) with ORF kustc1128-specific primers extended by a BamHI and a HindIII restriction site, respectively (acs-F5′GGATCCATGAATAAGACTGAAATAATAAATAAAC-3′ and acs-R5′AAGCTTATCTTCAAGTGTAGAAATATCTC-3′). Amplification was initiated with a denaturation step at 95 °C for 5 min and continued with an optimized amplification program of 30 cycles (1 min at 95 °C; 1 min at 52.5 °C; 3.5 min at 72 °C) with a final elongation step at 72 °C for 10 min. PCR products were cloned into the TopoTA vector (Invitrogen, UK) and transformed by heat shock into *E. coli* TOP10 (Invitrogen, UK) competent cells. Plasmids were isolated, digested with BamHI and HindIII and gel-purified with the Qiaex II Gel extraction kit (Qiagen Benelux, The Netherlands). The construct was ligated into the pET30a (Novagen, Germany) vector system.

### Mutant complementation

The constructed pET30a-*acs* vector was used to transform the *ackA*-*pta*-*acs* triple mutant *E. coli* AJW807 (Kumari et al. 1995), by the heat shock method. Progenies were selected on minimal medium (M63) supplemented with 10 mM Na-acetate, 0.02 % w/v glucose and 100 μg/ml ampicillin after induction with 75 μM isopropyl-β-d-thiogalactopyranoside (IPTG). After 3 days of incubation at 37 °C, colonies were transferred to Luria–Bertani medium plus 100 μg/ml ampicillin. Using the FlexiPrep™ kit (GE Healthcare Benelux, Belgium), plasmids were extracted to confirm the sequence accuracy by the Sanger method. Three colonies were selected from plate and resuspended in 1× PBS MgCl_2_ after washing. Cells were lysed by four sonification intervals of 15 s and used in the activity assay as described below.

### Expression and purification of recombinant ACS-like enzymes

The recombinant plasmids were used to transform *E. coli* JM109. Plasmids were extracted from the overnight culture to confirm sequence accuracy by Sanger sequencing. Flawless constructs were transformed into the expression host *E. coli* Rosetta™ 2 (DE3) (Novagen, Germany). Cells were grown at 37 °C in Luria–Bertani medium supplemented with 100 μg/ml kanamycin and 34 μg/ml chloramphenicol to an OD_600_ of approximately 0.6, and then the expression was initiated by the addition of 1 mM IPTG. After incubation for 3 h at 30 °C (final OD_600_ 1.0–1.2), cells were harvested by centrifugation from a total culture volume of 1 L. The His-tagged protein was purified with the Ni–NTA Spin kit (Qiagen Benelux, The Netherlands) according to the protocol of protein purification under native conditions from *E. coli* lysates using buffer NTI-10 and an additional disruption step in the French press for cell lysis. After confirming that the purified enzyme was the gene product of kustc1128 by MALDI-TOF MS analysis (Bruker Biflex III; Bruker Daltonics, USA), the eluted fractions were used for activity assays (described below).

### Preparation of *K. stuttgartiensis* whole cells

Cells were harvested by centrifugation (20 min, 4,000 rpm at 4 °C) and concentrated in 20 mM NaHCO_3_ buffer (pH 7.4). Negative controls were prepared by boiling cells for 15 min. The cells were used in an activity assay as described below.

### Enzyme assays and kinetic analysis

Assays were performed routinely at 37 °C in a total volume of 1 mL. The formation of acetyl-CoA from acetate, ATP and HSCoA was assessed by monitoring the Fe^3+^-acetyl hydroxamate complex formation from acetyl-CoA and hydroxylamine at 540 nm as adapted from Berg (1956). The assay mixture contained 145 mM Tris/HCl (pH 7.5), 10 mM MgCl_2_, 200 mM potassium acetate, 120 mM hydroxylamine hydrochloride (pH 7.5), 10 mM ATP, 0.47 mM HSCoA, 10 mM reduced glutathione and purified enzyme or whole cells. The reaction was stopped at 15 and 30 min, respectively, by adding 10 % TCA and 2.5 % FeCl_3_. This assay was used to determine the specific activity and apparent *K*_m_ values of the acetyl-CoA synthetase (AMP-forming) in acetate-grown cells of *E. coli*, the ACS activity in the fractions of the His-tag-purified enzyme preparations and the acetate conversion by whole cells of *K. stuttgartiensis*.

### Protein alignment and phylogenetic analysis

Sequences were aligned using the ClustalW multiple sequence alignment tool based on the Gonnet matrix options included in the MEGA 5.05 software (Tamura et al. 2011). A phylogenetic tree was constructed using both the neighbor-joining and the maximum likelihood algorithms of MEGA 5.05. Bootstrap values at the internal nodes were calculated from 1,000 iterations.

### Proteome and transcriptome

Materials and methods of proteome and transcriptome isolation and data analysis are described elsewhere (Kartal et al. 2011).

## Results and discussion

### Phylogeny and expression of genes encoding acetate-activating enzymes in *K. stuttgartiensis*

A thorough analysis of the *K. stuttgartiensis* genome assembly revealed seven protein-coding ORFs (>30 % identity) that were possibly involved in acetate metabolism as well as key enzymes of the acetyl-CoA pathway (Supplementary Table S1). Such a redundant repertoire of acetate- and acetyl-CoA-converting enzymes has not been observed in chemolithotrophic bacteria before.

**Fig. 1:**
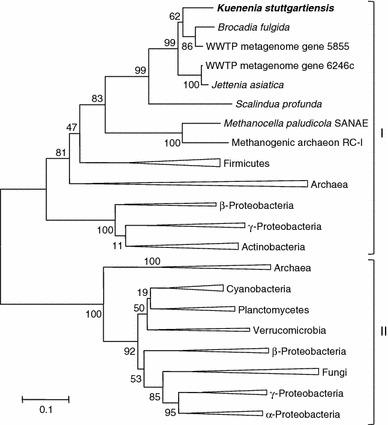
Neighbor-joining tree of phylogeny estimated by ClustalW included in the MEGA 5.05 software package, showing acetyl-CoA synthetase (AMP-forming) homologues with two different conserved domain architectures: cluster I and cluster II. Values at the internal nodes indicate bootstrap values based on 1,000 iterations

The most highly expressed ORF associated with acetate conversion in *K. stuttgartiensis* was kustc1128 encoding a putative AMP-forming acetyl-CoA synthetase (589 aa, calculated molecular mass 67 kDa; Table 1). Using the *K. stuttgartiensis* kustc1128 sequence as a template, a similar gene could also be identified in several other available anammox metagenomes (Gori et al. 2011; Harhangi et al. 2012; van de Vossenberg et al. 2012) suggesting a central role in anammox bacteria. The identities among the anammox ACS were >60 %. Phylogenetically, kustc1128 and other anammox homologues clustered at maximum identities of 57 % with representatives of the Archaea and Firmicutes (Fig. 1). These and several proteo- and actinobacterial sequences were affiliated to a larger cluster of acetyl-CoA synthetases that share a distinct domain structure (cluster I). The amino acid sequence differs significantly (<40 % identity) from the commonly described ACS (cluster II). A homologue of this cluster II could not be identified in *K. stuttgartiensis* after extensive analysis that makes kustc1128 the only ACS-like encoding gene.

**Table 1:** Relative gene expression and coverage in the proteome of potential acetate-activating enzymes in *K. stuttgartiensis*

Locus	Annotation	Gene expression^a^	Peptides^b^
kusta0048	Acetate-CoA ligase (ADP-forming); β-domain (*acdB*)	0.70	0
kustb2015	Acetate-CoA synthetase/acetate-CoA ligase	0.63	0
kustc0502	Acetate-CoA ligase (ADP-forming); α-domain (*acdA*)	0.46	0
kustc1128	Acetyl-CoA synthetase (*acsA*)	1.40	6 (13 %)
kuste3169	Acetyl-CoA synthetase (ADP-forming)	0.64	1 (2 %)
kuste3170	Hypothetical phsophotransacetylase protein	0.50	0
kuste3344	Phenylacetate-CoA ligase (*paaK*)	0.31	0

^a^Relative expression: (# reads × read length/ORF length, relative to overall coverage)

^b^Number of peptide hits (percentage coverage)

Closest hits were obtained (>60 %) to the ACS of hydrogenotrophic methanogens of the rice cluster I (RC-1), which use the enzyme for acetate assimilation (Erkel et al. 2006). Like the RC-1 archaeon, *K. stuttgartiensis* also encodes a putative vacuolar-type H^+^-translocating inorganic pyrophosphatase (kustd1836) that might function as a transmembrane proton pump (Serrano et al. 2004, 2007; Bielen et al. 2010). The pyrophosphate (PP_i_) released as a by-product of acetate activation, could theoretically be used to establish a proton motive force and thereby recover a quantity of the energy previously invested (Jetten et al. 1992).

### Mutant complementation tests

An *E. coli* strain (AJW807) deficient in all three pathways of acetate activation was used to determine the functionality of kustc1128 as an AMP-forming ACS.

The pET30a-containing mutant expressing kustc1128 upon induction with IPTG was able to grow on acetate as carbon source. Protein was isolated from a LB-grown *E. coli* AJW807 control group and from three different complemented mutants, grown on M63 supplemented with acetate. In the complemented mutant clones, the ACS enzyme had a specific activity of 57 nmol min^-1^ mg protein^−1^. This is in concert with earlier described ACS activity tests of the *ack* mutant of *E. coli* K12 (Brown et al. 1977). The mutant incapable of acetate activation showed significantly less activity (0.8 nmol min^−1^ mg protein^−1^). This remaining activity could be due to the background activity of phenylacetate-CoA ligase (Brunner et al. 1975) or long-chain acyl-CoA ligase (Kornberg and Pricer 1953). The expression of ORF kustc1128 restored the acetate-activating capacities in an *E. coli* *ackA*-*pta*-*acs* triple mutant, indicating its physiological role as an active AMP-forming ACS in *K. stuttgartiensis*.

### Substrate specificity and kinetic parameters

**Fig. 2:**
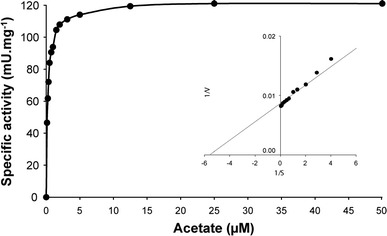
Rate dependence of the potential *K. stuttgartiensis* ACS activity at different acetate concentrations. The *inset* shows a plot of the reciprocal velocity against the reciprocal of the substrate concentration

The heterologous expression as a His-tagged protein allowed rapid purification of the *K. stuttgartiensis* ACS over a Ni–NTA column. The His-tagged ACS was loaded on 10 % SDS-PAGE (Supplementary Fig. S1). Only one prominent band was visible at the expected mass. MALDI-TOF MS analysis of the purified ACS after a trypsin digestion confirmed its identity as kustc1128 (Supplementary Fig. S1; sequence coverage 35.8 %). The purified enzyme was tested for substrate specificity and kinetic properties. Activity toward acetate was the highest among tested organic acids (130 ± 9 nmol min^−1^ mg protein^−1^; *n* = 6). The enzyme retained its activity over a wide pH range with an optimum around pH 7. Activity decreased with only 20 % between pH 6.5 and 8.5 (Supplementary Fig. S2). Relative to acetate, the propionate and formate conversion rates were reduced to 84 and 66 %, respectively, whereas butyrate (31 %) and isobutyrate (34 %) were converted at even lower rates (Table 2). The *K*_m_ for acetate was estimated at 0.2 mM that is comparable with *K*_m_ values of *E. coli, Haloarcula marismortui* and *Azotobacter aceti* ACS (O’Sullivan and Ettlinger 1976; Kumari et al. 1995; Bräsen and Schönheit 2005a) and well within the range of other described ACS enzymes (0.003–1.2 mM, (Bräsen et al. 2005b; Li et al. 2012) (Fig. 2). It has been reported previously that ACS could convert other organic substrates, in particular propionate, albeit with a significantly lower specific activity (Jetten et al. 1989; de Cima et al. 2007; Ingram-Smith and Smith 2007). The *K. stuttgartiensis* enzyme shows only a slightly reduced activity with propionate compared to acetate indicating a broad substrate range. Also, the conversion of longer substrates such as isobutyrate and butyrate is not a common characteristic and has been shown only for the archaeal ACS enzymes in *Archaeoglobus fulgidus* (ACS2) and *Pyrobaculum aerophilum* (Bräsen et al. 2005; Ingram-Smith and Smith 2007). It is hypothesized that the broad substrate specificity is established by a substitution in one of the four conserved residues in the acetate-binding pocket that determines the specificity of the acyl-substrate (Ingram-Smith et al. 2006b). Based on sequence comparison, the Ile^312^ in the *K. stuttgartiensis* ACS is replaced by Val, a trait conserved among the described organisms sharing similar catalytic properties.

**Table 2:** Specific activity of the purified ACS-like enzyme with different organic acids

Organic acid	Rate (nmol min^−1^ mg protein^−1^)	% of rate with acetate
Acetate	130.3	100
Propionate	114.9	88
Formate	84.8	65
Butyrate	40.5	31
Iso-butyrate	39.1	30

### Acetate conversion in *Kuenenia stuttgartiensis*

**Fig. 3:**
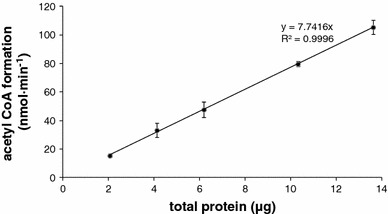
Formation of acetyl-CoA from potassium acetate in response to the addition of different amounts of whole *K. stuttgartiensis* cells

That anammox bacteria can use organic acids as electron acceptor has been shown previously (Kartal et al. 2007b, 2008). The fate of those organics is until now still speculative, but all known pathways of acetate or propionate conversion proceed via acetyl-CoA, which would also be the end product of carbon fixation in anammox bacteria. As incorporation of acetate-derived carbon has not be shown yet, whole cells of *K.* *stuttgartiensis* were incubated with acetate ATP, and HSCoA and the conversion into acetyl-CoA were determined by measuring the Fe^3+^-acetyl hydroxamate complex formation. The rate of acetyl-CoA formation was significantly higher than for the complemented *E. coli* mutant (7.7 μmol min^−1^ mg^−1^) (Fig. 3). The conversion of acetate to acetyl-CoA increased linearly with the amount of cells added, whereas boiled cells did show any activity. Considering this assay relies on total protein concentrations, this rate could very well fit with that of the heterologously expressed, His-tag purified enzyme.

In the present study, we could show that acetate could be activated by kustc1128, an *acs*-like protein, as well as whole cells of *K. stuttgartiensis* suggesting that indeed the reductive acetyl-CoA pathway was used by anammox bacteria as previously suggested. Such acetate activation could also lead to the direct incorporation of acetate into cell biomass by anammox bacteria.

Additionally, the PP_i_ released upon the formation of acetyl-CoA could be used to translocate protons by an H^+^-translocating pyrophosphatases building up a proton motive force over the anammoxosomal membrane, which is central to the anammox catabolism (Kartal et al. 2011). Recently, it was shown that the ATP-consuming reaction of ACS could be coupled to ATP-producing processes, a possibility that gives interesting perspectives regarding further research on anammox carbon metabolism (Mayer et al. 2012).

## Electronic supplementary material

Below is the link to the electronic supplementary material.

Supplementary material 1 (PDF 85 kb)

## References

[CR1] Arrigo KR (2005). Marine microorganisms and global nutrient cycles. Nature.

[CR2] Berg P (1956). Acyl adenylates—enzymatic mechanism of acetate activation. J Biol Chem.

[CR3] Bielen AAM, Willquist K, Engman J, van der Oost J, van Niel EWJ, Kengen SWM (2010). Pyrophosphate as a central energy carrier in the hydrogen-producing extremely thermophilic *Caldicellulosiruptor saccharolyticus*. FEMS Microbiol Lett.

[CR4] Bräsen C, Schönheit P (2005). AMP-forming acetyl-CoA synthetase from the extremely halophilic archaeon *Haloarcula marismortui*: purification, identification and expression of the encoding gene, and phylogenetic affiliation. Extremophiles.

[CR5] Bräsen C, Urbanke C, Schönheit P (2005). A novel octameric AMP-forming acetyl-CoA synthetase from the hyperthermophilic crenarchaeon *Pyrobaculum aerophilum*. FEBS Lett.

[CR6] Brown TDK, Jones-Mortimer MC, Kornberg HL (1977). The enzymic interconversion of acetate and acetyl-coenzyme A in *Escherichia coli*. J Gen Microbiol.

[CR7] Brunner R, Rohr M, Hash JH (1975) Phenacyl: coenzyme A ligase. Methods Enzymol. Academic Press, pp 476–481 10.1016/0076-6879(75)43107-x 237212

[CR8] de Cima S, Rúa J, del Valle P, Busto F, Baroja-Mazo A, de Arriaga D (2007). Different stabilities of two AMP-forming acetyl-CoA synthetases from *Phycomyces blakesleeanus* expressed under different environmental conditions. J Biochem.

[CR9] Erkel C, Kube M, Reinhardt R, Liesack W (2006). Genome of rice cluster I archaea—the key methane producers in the rice rhizosphere. Science.

[CR10] Gori F, Green-Tringe S, Kartal B, Machiori E, Jetten MSM (2011). The metagenomic basis of anammox metabolism in *Candidatus* ‘Brocadia fulgida’. Biochem Soc Trans.

[CR11] Güven D (2005). Propionate oxidation by and methanol inhibition of anaerobic ammonium-oxidizing bacteria. Appl Environ Microbiol.

[CR12] Harhangi HR (2012). Hydrazine synthase, a unique phylomarker to study the presence and biodiversity of anammox bacteria. Appl Environ Microbiol.

[CR13] Ingram-Smith C, Smith KS (2007). AMP-forming acetyl-CoA synthetases in Archaea show unexpected diversity in substrate utilization. Archaea.

[CR14] Ingram-Smith C, Martin SR, Smith KS (2006). Acetate kinase: not just a bacterial enzyme. Trends Microbiol.

[CR15] Ingram-Smith C, Woods BI, Smith KS (2006). Characterization of the acyl substrate binding pocket of acetyl-CoA synthetase. Biochemistry.

[CR16] Jetten MSM, Stams AJM, Zehnder AJB (1989). Isolation and characterization of acteyl-coenzyme A synthetase from *Methanothrix soehngenii*. J Bacteriol.

[CR17] Jetten MSM, Fluit TJ, Stams AJM, Zehnder AJB (1992). A fluoride-insensitive inorganic pyrophosphatase isolated from *Methanothrix soehngenii*. Arch Microbiol.

[CR18] Juretschko S (1998). Combined molecular and conventional analyses of nitrifying bacterium diversity in activated sludge: *Nitrosococcus mobilis* and *Nitrospira*-like bacteria as dominant populations. Appl Environ Microbiol.

[CR19] Kartal B (2007). Anammox bacteria disguised as denitrifiers: nitrate reduction to dinitrogen gas via nitrite and ammonium. Environ Microbiol.

[CR20] Kartal B (2007). *Candidatus* ‘Anammoxoglobus propionicus’ a new propionate oxidizing species of anaerobic ammonium oxidizing bacteria. Syst Appl Microbiol.

[CR21] Kartal B (2008). *Candidatus* ‘Brocadia fulgida’: an autofluorescent anaerobic ammonium oxidizing bacterium. FEMS Microbiol Ecol.

[CR22] Kartal B (2011). Molecular mechanism of anaerobic ammonium oxidation. Nature.

[CR23] Kornberg A, Pricer WE (1953). Enzymatic synthesis of the coenzyme A derivatives of long chain fatty acids. J Biol Chem.

[CR24] Kumari S, Tishel R, Eisenbach M, Wolfe AJ (1995). Cloning, characterization, and functional expression of *acs*, the gene which encodes acetyl-coenzyme A synthetase in *Escherichia coli*. J Bacteriol.

[CR25] Lam P, Kuypers MMM (2011). Microbial nitrogen cycling processes in oxygen minimum zones. Ann Rev Mar Sci.

[CR26] Li R, Gu J, Chen P, Zhang Z, Deng J, Zhang X (2012). Purification and characterization of the acetyl-CoA synthetase from *Mycobacterium tuberculosis*. ABBS.

[CR27] Mayer F (2012). AMP-forming acetyl coenzyme a synthetase in the outermost membrane of the Hyperthermophilic Crenarchaeon *Ignicoccus hospitalis*. J Bacteriol.

[CR28] O’Sullivan J, Ettlinger L (1976). Characterization of the acetyl-CoA synthetase of *Acetobacter aceti*. Biochim Biophys Acta.

[CR29] Schouten S (2004). Stable carbon isotopic fractionations associated with inorganic carbon fixation by anaerobic ammonium-oxidizing bacteria. Appl Environ Microbiol.

[CR30] Serrano A, Perez-Castineira JR, Baltscheffsky H, Baltscheffsky M (2004). Proton-pumping inorganic pyrophosphatases in some archaea and other extremophilic prokaryotes. J Bioenerg Biomembr.

[CR31] Serrano A, Perez-Castineira JR, Baltscheffsky M, Baltscheffsky H (2007). H^+^-PPases: yesterday, today and tomorrow. IUBMB Life.

[CR32] Starai VJ, Escalante-Semerena JC (2004). Acetyl-coenzyme A synthetase (AMP forming). Cell Mol Life Sci.

[CR33] Strous M, Heijnen JJ, Kuenen JG, Jetten MSM (1998). The sequencing batch reactor as a powerful tool for the study of slowly growing anaerobic ammonium-oxidizing microorganisms. Appl Microbiol Biotechnol.

[CR34] Strous M (2006). Deciphering the evolution and metabolism of an anammox bacterium from a community genome. Nature.

[CR35] Tamura K, Peterson D, Peterson N, Stecher G, Nei M, Kumar S (2011). MEGA5: molecular evolutionary genetics analysis using maximum likelihood, evolutionary distance, and maximum parsimony methods. Mol Biol Evol.

[CR36] van de Vossenberg J et al (2012) The metagenome of the marine anammox bacterium ‘*Candidatus* Scalindua profunda’ illustrates the versatility of this globally important nitrogen cycle bacterium. Environ Microbiol published online:doi:10.1111/j.1462-2920.2012.02774.x 10.1111/j.1462-2920.2012.02774.x PMC3655542 22568606

[CR37] Wolfe AJ (2005). The acetate switch. Microbiol Mol Biol Rev.

