# Supplementary material for: Genome analysis and heterologous expression of acetate-activating enzymes in the anammox bacterium Kuenenia stuttgartiensis
Source: Arch Microbiol. 2012 Jul 1;194(11):943–8. doi: 10.1007/s00203-012-0829-7 (PMC3477478; doi:10.1007/s00203-012-0829-7)
Supplement: Supplementary file 1 — Supplementary material 1 (PDF 85 kb) [file 203_2012_829_MOESM1_ESM.pdf]

Supplementary Table S1 Description and characterization of *K. stuttgartiensis* ORFs possibly participating in acetate or acetyl-CoA interconverting reactions.

***K. stuttgartiensis* genome**				**Blast Output**		
Query	Annotation	Length	Domains	Organism	Annotation	E-value
kustb0215	acetyl-coenzyme A synthetase/acetate-CoA ligase (caiC)	528aa	COG0318	uncultured delta proteobacterium ADI17226.1	acyl-CoA synthetases (AMP-forming)/AMP-acid ligases II	5.00E-88
kustc0502	acetyl-CoA synthetase (ADP-forming); α- domain (acdA)	462aa	TIGR02717	*Methanosarcina mazei* Go1 NP_632382.1	acetyl-CoA synthetase, α-subunit	1.00E-101
kustd1545	CO dehydrogenase/acetyl-CoA synthase; α-subunit	727aa	PRK09529	*Ammonifex degensii* KC4 YP_003238347	CO dehydrogenase/acetyl-CoA synthase complex; β-subunit	0
kustd1546	CO dehydrogenase/acetyl-CoA synthase; β-subunit	653aa	TIGR01702 COG1151 pfam03063	*Ammonifex degensii* KC4 YP_003238346	CO dehydrogenase, catalytic subunit	2.00E-177
kuste3170	hypothetical phosphotransacetylase protein	356aa	COG0857	*Desulfovibrio desulfuricans* G20 ABB38502.1	cobyrinic acid a,c-diamide synthase family protein	6.00E-67
kuste3344	phenylacetate-CoA ligase (paaK)	435aa	COG1541	*Chitinophaga pinensis* DSM 2588 YP_003121574.1	F390 synthetase-like protein	1.00E-79
kuste4610	CO dehydrogenase/acetyl-CoA synthase; β-subunit	658aa	PF03063	*Thermincola* sp. JR YP_003640687	carbon-monoxide dehydrogenase, catalytic subunit	2.00E-144
kusta0048	acetate-CoA ligase (ADP-forming); β-domain (acdB)	242aa	COG1042	*Cand.* 'Korarchaeum cryptofilum OPF8' YP_001736558.1	hypothetical protein Kcr_0115	5.00E-64
kuste3169	acetyl-CoA synthetase (ADP-forming)	753aa	TIGR02717 COG1042	*Chloroflexus aurantiacus* J10fl YP_001637486.1	acetyl CoA synthetase; α-subunit	0
kustc1128	acetyl-CoA synthetase (acsA)	589aa	PRK04319	*Methanocella paludicola* SANAE YP_003355474.1	acetyl-CoA synthetase	0

Supplementary Figure S1: The Ni-NTA purified acetyl CoA synthetase was separated on a 10% SDS PAGE (right bottom). The band at about 65 kDa was excised and trypsinised. The trypsin digest was analyzed by Maldi tof MS (spectrum below) to confirm the identity of the ACS. The ACS sequence with the identified parts is showed at the right part.

MHHHHHHSSG LVPRGSGMKE TAAAKFERQH MDSPDLGTDD DDKAMADIGS
MNKTEIINKH PEAFNLICYE DQHKNFSWET VKKELGVGGN KVNIAYEAID
KHATTWRKNK VALYWEGSDG THLKYTFQEL KILSDKCANM LQSLGVGKGD
RVFLFLPRLP ELFINMIAIA KLGAISGPMF SAFGPDAVRD RLQNSEAKVL
ITTPELKERV DAVLWELPKL ERIVLVSVKE DYELEEGNVC YKTLMKDAPE
RFEMEWMDME DPLYLLYTSG TTGKPKGITH VHNDMISYYI TTKWSLDLRD
DDIYWCTADP GWVTGMVYGM WGPWLNGVSM YIYDGRFDVN KWYEAIQSYK
ITVWYTAPTA LRMLMKSGDY LVAQYDLESL RYICSVGEPL NPEVIKWGMN
VYNLPIHDTW WQTETGSIMI ANYPCIPIKP GSMGKPFPGI KAAIIDSEGN
ELPDGHHGIL ALKPGWPSML RKVWGDEGRF NEYFNITGWY TTGDTAYKDE
EGYFWFVGRA DDVINTSGHR VGPFEVESAL LEHRAVAEAG VIGKPDPERG
EIIKAFIALK EGFKPSSELG EEIKKFIKHH LAAHAYPREI EFCENLPKTR
SGKIMRRLLK AKDLGLPTGD ISTLEDKLAA ALEHHHHHH

K. stuttgartiensis Acs (underlined)

Identified by Maldi-tof MS (in red)

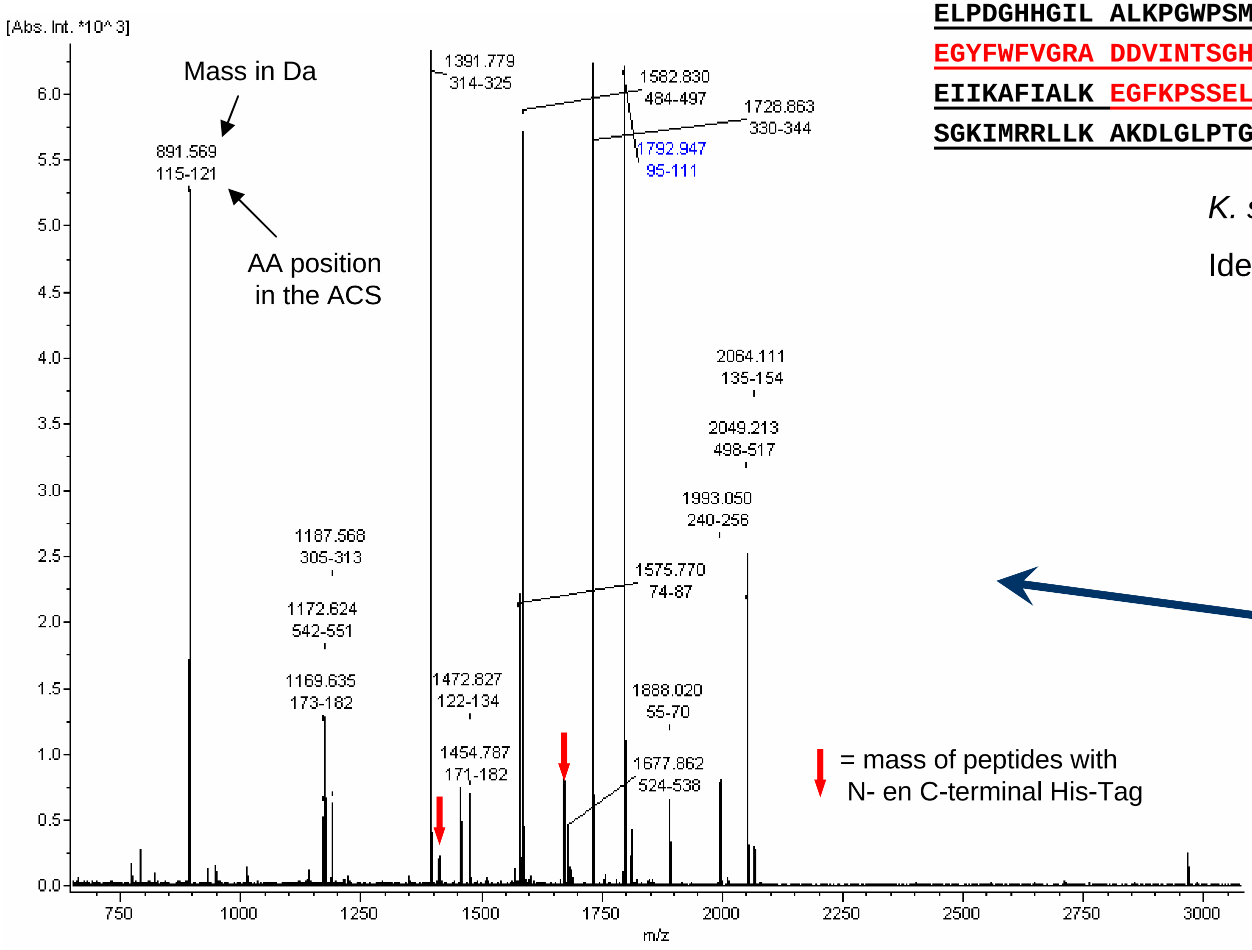

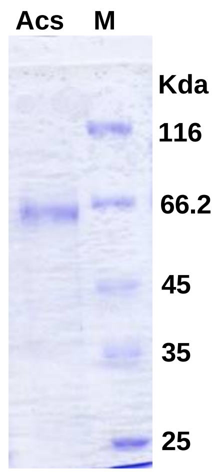

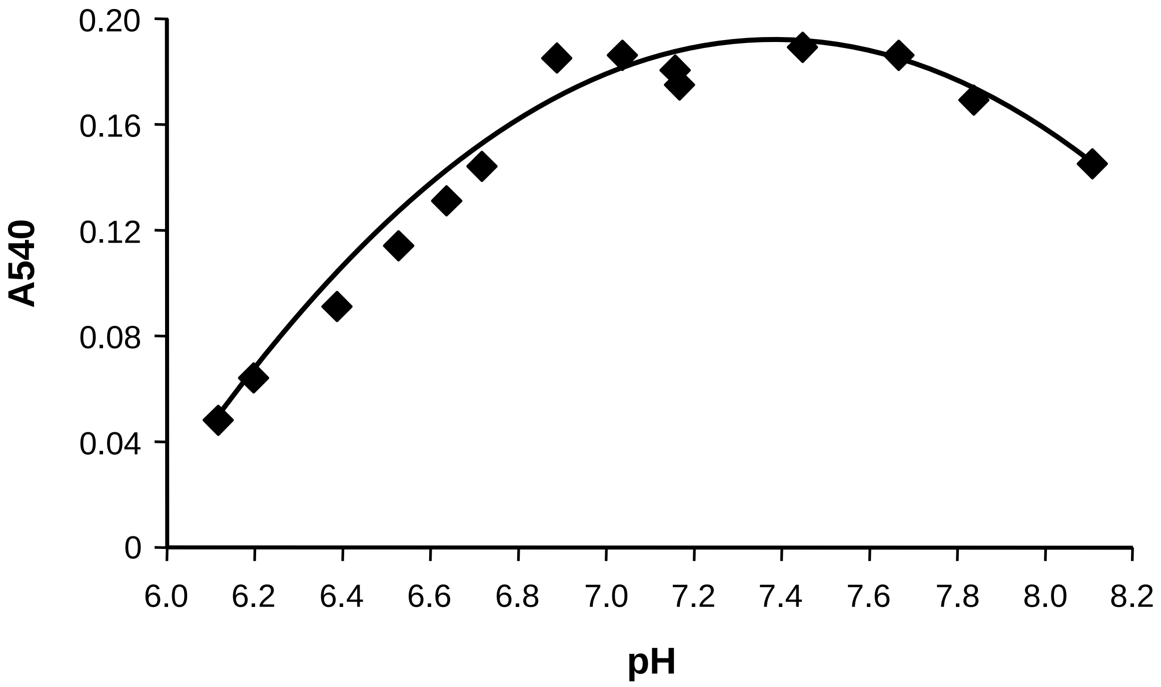

Supplementary Fig. S2 Effect of pH on the activity of the heterologous expressed acetyl CoA synthetase of *K. stuttgartiensis* (kustc1128).
